# Editorial: Tumor microenvironment targeted nanomedicine: a feasible strategy for cancer imaging and theranostics

**DOI:** 10.3389/fonc.2023.1228910

**Published:** 2023-06-07

**Authors:** Yiming Xu, Dawei Jiang, Weiyu Chen

**Affiliations:** ^1^ Department of Respiratory and Critical Care Medicine, The Fourth Affiliated Hospital, Zhejiang University School of Medicine, Yiwu, China; ^2^ Union Hospital, Tongji Medical College, Huazhong University of Science and Technology, Wuhan, Hubei, China; ^3^ International Institutes of Medicine, The Fourth Affiliated Hospital of Zhejiang University School of Medicine, Yiwu, Zhejiang, China

**Keywords:** tumor microenvironment (TME), nanomedicine, cancer imaging, theranostics, tumor-targeted delivery

Tumor microenvironment (TME) has been widely recognized as a critical part of cancer development. TME consists of all the non-cancerous cells, including immune cells, fibroblasts, endothelial cells, neurons, adipocytes, as well as some non-cellular components such as extracellular matrix (ECM), cytokines and chemokines. The characteristics of TME and the cross-talk between TME and cancer cells contribute greatly to all stages of tumorigenesis and cancer progression (1). Thus, targeting TME for cancer imaging and therapies is a promising field for cancer research. However, despite the advances made in recent years, many clinical trials targeting TME have failed due to unsatisfactory therapeutic efficacy in cancer patients (2). The main reasons may contribute to the shortage of a deep understanding of the intricate mechanisms of TME and effective adjuvant techniques. In recent years, nanotechnology has been rapidly developed and demonstrated great application value for treating various diseases, including cancer (3). Since the physiochemical features and ease of synthetic design of nanomaterials, nanomedicine could be designed as cancer therapeutic regimens according to the characteristics of TME (**Figure 1**).

**Figure 1:**
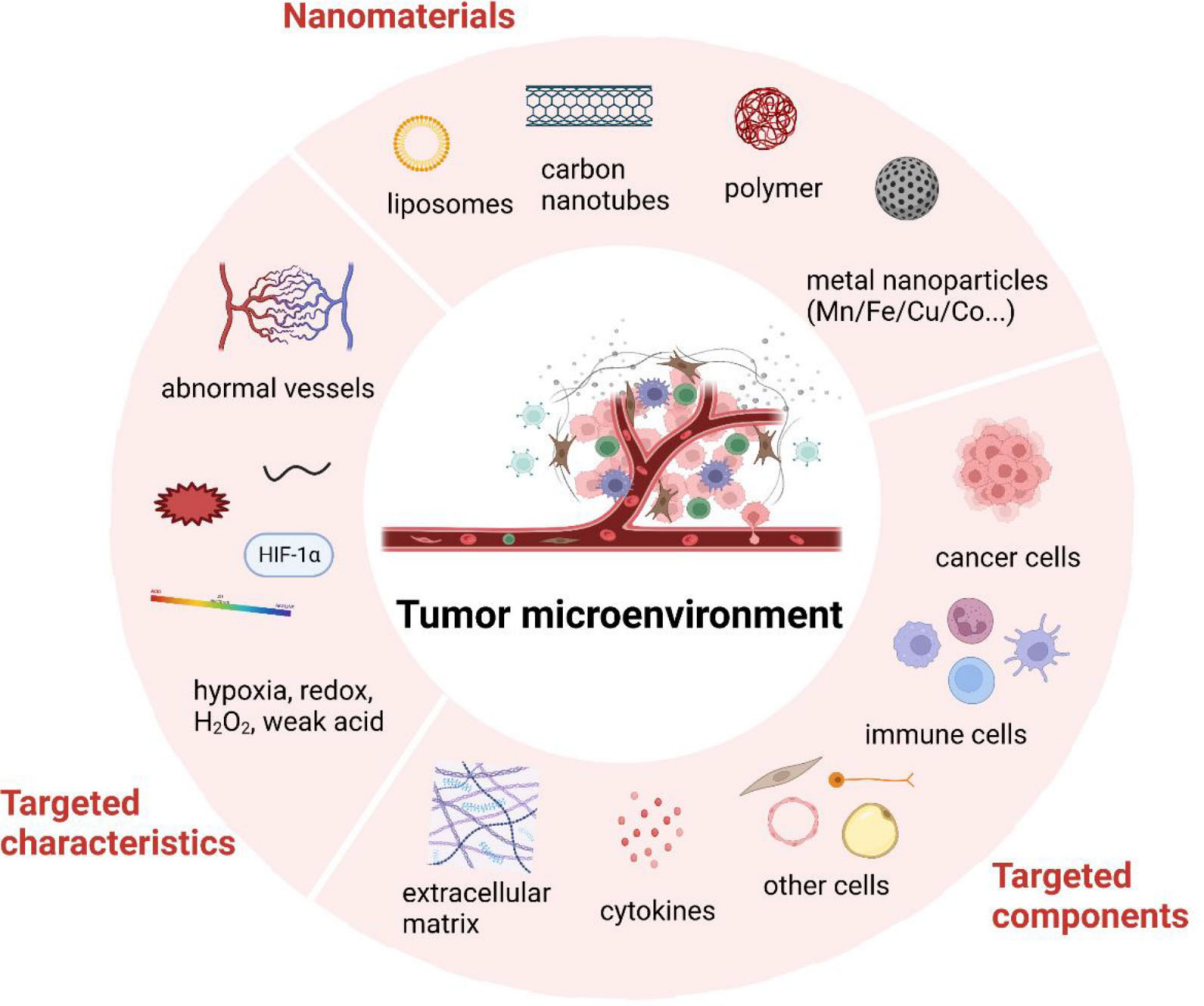
Brief illustration of tumor microenvironment and nanomedicine targeted strategies. Created with BioRender.com.

This Research Topic, “*Tumor Microenvironment Targeted Nanomedicine: A Feasible Strategy for Cancer Imaging and Theranostics*,” contains five most-recent review articles. Each study has systematically reviewed certain nanotechnologies, showing great value in various clinical applications for treating cancer.

Immune cells occupy a large part of TME with both innate (macrophages, neutrophils, dendritic cells, innate lymphoid cells, myeloid-derived suppressor cells, and natural killer cells) and adaptive immune cells (T cells and B cells) (4). In the past few decades, cancer immunotherapy has progressed much, in which immune checkpoint inhibitors are included in the first-line treatments. However, the suppressive immune microenvironment and insufficient immune response rate greatly restrain the therapeutic efficacy. Notably, the activation of STING signaling pathway with STING agonists shows great potential in turning the “cold” tumor into “hot” tumor, thus enhancing the antitumor immune response in the TME (Chen et al.). By loading different STING agonists such as cGAMP, SR-717, MSA2, PC7A or co-stimulating with other immune agents, designed nanomedicine could act as a prospective platform for delivering and amplifying the effectiveness of STING agonists for cancer immunotherapy (Chen et al.). In addition, the abnormal tumor vessels in TME serve as another immunosuppressive factor (5). Tumor angiogenesis is a complex process requiring the participation of tumor cells, endothelial cells, immune cells, ECM, cytokines, etc. The abnormal structure and function of tumor vasculature contribute greatly to cancer progression as it is one of the critical reasons for tumor hypoxia and low PH, inefficient drug delivery, and reduced immune cell infiltration (Xiao et al.). Nanomaterial-based anti-angiogenic therapy has exhibited prior advantages to traditional anti-angiogenic drugs, showing less side effects and drug resistance. To be more specific, Xiao et al. comprehensively overviewed the characteristics and molecular mechanisms of abnormal tumor vasculature and discussed the connection with tumor immune microenvironment, further summarizing the recent advances of nanomedicines for targeting tumor angiogenesis, which influenced tumor immunotherapy.

TME illustrates unique features, such as low pH, hypoxia, high H_2_O_2_, high GSH, etc., contributing to cancer malignancy. Notably, these distinct characteristics also enable nanomedicine to target tumors and mediate effective therapy *via* TME-responsive designs. For example, hypoxia-responsive nanomedicines (e.g., hypoxia-targeted, hypoxia-alleviated, and hypoxia-triggered nanomaterials) can enhance the therapeutic efficacy by smartly hypoxia-specific drug releasing (Xia et al.). Besides, combined with certain nano-contrast agents for ultrasound, magnetic resonance imaging (MRI), or positron emission tomography (PET) imaging, these TME-responsive nanomaterials may offer earlier and more precise tumor detection than traditional tumor imaging agents.

Moreover, these well-designed nanomedicine for cancer imaging and theranostics can provide better biosafety than conventional strategies. For example, gadolinium (Gd)-based contrast agents are most frequently used in clinical MRI. However, the safety of Gd-based contrast agents has raised great concerns due to some severe side effects. Various recent studies have demonstrated that manganese (Mn)-based nanomaterials can also be applied for MRI with great biocompatibility and accurate cancer diagnosis (Liu and Rong). In addition, Mn-based hybrid nanomaterials show great responsiveness to TME. Thus, such nano-platform can assist drug delivery and traditional cancer therapies by serving as imaging-guided agents, giving a prospective sight for developing cancer precision medicine (Liu and Rong). Another kind of nanomaterial, carbon nanotubes (CNTs), characterized with nanosized hollow tube-shaped structures with unique physical-chemical characteristics ranging from strong near-infrared absorbance to good photothermal performance, has shown broad application potential for drug delivery, cancer imaging and cancer photothermal therapy as well (Zhang et al.). However, CNTs exposure may be related to malignant mesothelioma development and lung damage (Zhang et al.). Thus, it well reminds us that every coin has two sides. Undeniably, nanomedicine has shown huge potential in cancer diagnosis and treatment, but there is still a long way.

In summary, this Research Topic presents these most recent review articles and sums up the current advancement of TME-targeted nanomedicine from several perspectives. Further studies that reveal the underlying mechanisms of TME and nanotechnology innovation would be critical for clinically used in cancer imaging and theranostics.

## Author contributions

Conceptualization, YX, DJ and WC; Writing—original draft preparation, YX and WC; Writing—review and editing, DJ and WC. All authors have read and agreed to the published version of the manuscript.
